# Domain landscapes of somatic mutations in cancer

**DOI:** 10.1186/1471-2164-13-S4-S9

**Published:** 2012-06-18

**Authors:** Nathan L Nehrt, Thomas A Peterson, DoHwan Park, Maricel G Kann

**Affiliations:** 1 Department of BiologicalSciences, University of Maryland, Baltimore County, 1000 Hilltop Circle, Baltimore, MD 21250, USA; 2 Division of Imaging and Applied Mathematics, OSEL, CDRH, U.S. Food and Drug Administration, 10903 New Hampshire Avenue, Silver Spring, MD 20993, USA; 3 Department of Mathematics and Statistics, University of Maryland, Baltimore County, 1000 Hilltop Circle, Baltimore, MD 21250, USA

## Abstract

**Background:**

Large-scale tumor sequencing projects are now underway to identify genetic mutations that drive tumor initiation and development. Most studies take a gene-based approach to identifying driver mutations, highlighting genes mutated in a large percentage of tumor samples as those likely to contain driver mutations. However, this gene-based approach usually does not consider the position of the mutation within the gene or the functional context the position of the mutation provides. Here we introduce a novel method for mapping mutations to distinct protein domains, not just individual genes, in which they occur, thus providing the functional context for how the mutation contributes to disease. Furthermore, aggregating mutations from all genes containing a specific protein domain enables the identification of mutations that are rare at the gene level, but that occur frequently within the specified domain. These highly mutated domains potentially reveal disruptions of protein function necessary for cancer development.

**Results:**

We mapped somatic mutations from the protein coding regions of 100 colon adenocarcinoma tumor samples to the genes and protein domains in which they occurred, and constructed topographical maps to depict the “mutational landscapes” of gene and domain mutation frequencies. We found significant mutation frequency in a number of genes previously known to be somatically mutated in colon cancer patients including *APC*, *TP53* and *KRAS*. In addition, we found significant mutation frequency within specific domains located in these genes, as well as within other domains contained in genes having low mutation frequencies. These domain “peaks” were enriched with functions important to cancer development including kinase activity, DNA binding and repair, and signal transduction.

**Conclusions:**

Using our method to create the domain landscapes of mutations in colon cancer, we were able to identify somatic mutations with high potential to drive cancer development. Interestingly, the majority of the genes involved have a low mutation frequency. Therefore, themethod shows good potential for identifying rare driver mutations in current, large-scale tumor sequencing projects. In addition, mapping mutations to specific domains provides the necessary functional context for understanding how the mutations contribute to the disease, and may reveal novel or more refined gene and domain target regions for drug development.

## Background

The advent of high-throughput, whole-genome DNA sequencing has enabled the evaluation of normal and tumor tissue samples from hundreds of patients in a single study, revealing both germline and somatic mutations with potential involvement in cancer susceptibility, initiation and development. However, distinguishing the handful of somatic mutations expected to initiate and maintain tumor growth, so-called driver mutations, from mutations that play no role in cancer development, passenger mutations, is still a major hurdle to fully understanding the mechanisms of the disease and to the design of more effective treatments. Most current, state-of-the-art studies take a gene-centric approach to the problem [1-6], identifying potential driver mutations as those that occur in genes mutated in a high percentage of the tumor samples. A pathway analysis typically follows to add functional context to the mutated genes. For example, mutations in the *APC* gene have been shown to be highly prevalent in colorectal tumors [1,2,7]. Unfortunately, this approach is limited to a small subset of genes and inherently disregards gene mutations occurring in a low percentage of tumor samples. Identifying rare mutations at the gene level, those that do not recur in the same gene in many patients, with high functional relevance to the oncogenic process is extremely difficult using current gene-centric approaches. This is indeed one of the most crucial problems in the fight against cancer today (http://provocativequestions.nci.nih.gov).

Furthermore, gene-centric approaches to classifying driver and passenger mutations make no distinction between mutations in different sites on the gene, disregarding important information about the functional context of the site of the mutation. A recent study by Vidal’s team demonstrated the potential of gene-centric approaches to mischaracterize mutations [8]. The authors showed that changes causing a complete knockout of a protein (node removal) are often phenotypically distinct from mutations that disrupt specific regions of the proteins thereby eliminating any interaction(s) in which the protein participates (edgetic perturbations). In particular, the authors emphasized the importance of taking into account the modularity of proteins when studying mutation-phenotype relationships, showing several examples where mutations in the same protein but in different protein domains, which are protein regions conserved within and across species [9], produce distinct disease phenotypes. This result also demonstrates how pathway analyses of mutated genes can potentially provide an incomplete picture of the functional implications of mutations at the gene level. Distinct interactions for a protein in the pathway can either be preserved or disrupted depending on whether or not mutations affect the specific domain mediating the interaction.

In this study, we introduce a new approach for the analysis of cancer somatic mutations based on the study of these mutations at the protein domain level. We argue that since protein domains define the structural and functional units of the proteins, mapping mutations not only to the genes in which they occur, but also to individual protein domains, adds functional information critical to the accurate assessment of the impact of the mutations. Analysis of the positions of individual oncogenic mutations discovered from several independent studies has revealed significant clustering of these mutations at specific positions within the catalytic domain of several protein kinases [1,11,12]. Other studies specifically designed to show the clustering of mutations at distinct positions within protein domains have revealed numerous other domain positions highly mutated across a variety of disease types [12,13]. In a functional analysis of candidate colon cancer genes identified by Sjöblom *et al.* in 2006, significant enrichments of proteins containing the MH1 and MH2 domains were found [1]. However, as later noted by Chittenden *et al.*, whereas 70% of the mutations in MH2-containing proteins fell within the MH2 domain, the enrichment of the MH1 domain turned out to be misleading as further analysis revealed that none of mutations in the MH1-containing proteins occurred inside of the MH1 domain itself [14]. Similarly, many other distinct domains have been shown to be significantly enriched in cancer-associated genes including kinase domains and domains involved in transcriptional regulation and DNA maintenance and repair [15]. Domain enrichment analysis, however, is commonly performed after a significant set of genes has been identified, and does not consider whether mutations in the genes actually occur inside the enriched domains. This approach can result in misleading assumptions about domain associations to cancer.

Our approach for identifying mutations relevant to cancer development specifically maps somatic mutations to the individual domains in which they occur, resulting in a more accurate measure of enrichment of mutations at the domain level. By mapping mutations to individual domains, our method avoids potentially misleading conclusions from gene-based domain enrichment analyses and provides an inherent functional explanation for how the mutations contribute to disease. We performed an exome-wide study of somatic mutations, including single nucleotide variants (SNVs) and short insertions and deletions (indels), from 100 colon adenocarcinoma patients obtained from The Cancer Genome Atlas (TCGA) project [16]. To implement our approach, mutations in each domain are aggregated from all human proteins containing the domain. As a result we aggregate a wide range of human proteins, from those from the same protein families sharing high sequence similarity to highly dissimilar proteins sharing only the domain in which the mutations are present. By doing so, our domain-centric method can also reveal novel gene candidates for involvement in cancer development, through the identification of a highly mutated domain shared with other genes known to be significant in cancer.

We also introduce new terminology to describe the distribution of somatic mutations at the protein domain level: the “domain mutational landscape” consisting of a topographic representation of mutation frequencies within individual protein domains from whole-genome, cancer sequencing studies, and “domain peaks” defined as protein domains mutated at high-frequency in tumor genomes of the same or different tumor types. Sjöblom and collaborators performed the first large-scale analysis of breast and colorectal cancer mutations [1]. Using a small sample of individual tumors, they identified 191 candidate genes (CAN genes) significantly mutated in breast and colorectal tumor samples. Wood *et al.* later followed up the study using a larger set of transcripts and revised statistics for identifying significantly mutated genes [2]. The authors identified an additional 89 CAN genes, and postulated that the genomic landscape of cancer is composed of a few commonly mutated gene “mountains”, including* APC*, *KRAS*, *TP53* and others for colorectal cancer, but is dominated by a larger number of infrequently mutated gene “hills”. Here, we compare the gene-based mutational landscape of a much larger set of colon tumor samples to the landscape revealed by Wood *et al.*, and show significant clustering of mutations in many of the previously identified CAN genes.

In addition, we also describe the domain mutational landscape of colon cancer, and demonstrate how this landscape reveals major properties that cannot be revealed by gene-based landscapes. We show how highly mutated domain peaks can be missed by gene-centric methods when the individual genes containing the domains are not mutated at high frequencies. We also show how focusing only on significantly mutated genes can miss instances where mutations occurring within a shared domain are actually the more relevant functional contributors to the cancer. Therefore, due to its ability to identify and functionally characterize somatic mutations with high potential to drive cancer development, we expect our novel domain-centric method to become an integral tool for the analysis of data from future large-scale cancer sequencing studies.

## Results

In this study, we compared the gene and domain mutational landscapes of somatic mutations present in tumor samples from 100 colon adenocarcinoma patients participating in the TCGA project. Mutations in protein coding genes such as single nucleotide variants causing amino acid changes (nonsynonymous SNVs or nsSNVs), short insertions or deletions causing a shift in the reading frame (frame shift mutations) and mutations causing the gain or loss of a stop codon (stop-gain or stop-loss mutations) are generally expected to be the most likely candidates for driving disease development. Therefore, we identified all somatic mutations of these types in addition to nonframeshift insertions and deletion mutations occurring within the protein coding regions of the tumor samples (see Methods for additional details). In total, 21,572 mutations were identified in the 100 samples, yielding an average of 215 mutations per patient. Approximately 80% of the mutations were nsSNVs, 12% were frame shift mutations, 7% were stop-gain mutations, 0.15% were stop-loss mutations, and 1% were nonframeshift insertion mutations (Table 1). In addition, almost half (49.4%) of all mutations occurred inside of annotated protein domain regions. For comparison to another cancer type, we also created the gene and domain mutational landscapes for 522 breast invasive carcinoma patients also participating in the TCGA project. Despite having a larger number of mutations (25,807), the breast cancer patients had a lower average mutation count (49.7), but the distribution of mutation types and number of mutations inside of domain regions were roughly similar to the colon cancer set (Additional file 1 - Table S1).

**Table 1:** Mutation counts for colon cancer

Total patients	100
Total mutations	21,572

Total nonsynonymous SNVs	17,174 (79.6%)

Total frameshift insertions	2,527 (11.7%)

Total nonframeshift insertions	239 (1.1%)

Total frameshift deletions	5 (0.0%)

Total nonframeshift deletions	0 (0.0%)

Total stop-loss SNVs	33 (0.2%)

Total stop-gain SNVs	1,594 (7.4%)

Mutations in domain regions	10,647 (49.4%)

Average mutations per patient	216 (± 552)

Number of mutations per patient	21-4,880

Summary of somatic mutations occurring in the exomes of 100 colon cancer tumor samples. Synonymous SNVs and variants present in dbSNP (release 130) were removed due to their low likelihood of being driver mutations.

Here, we show results of mapping mutations not only to individual genes, but also to the specific protein domains in which they occurred. We also constructed the domain-based mutational landscape for colon cancer from a set of 100 tumor samples.

### Gene mutation landscapes

**Figure 1:**
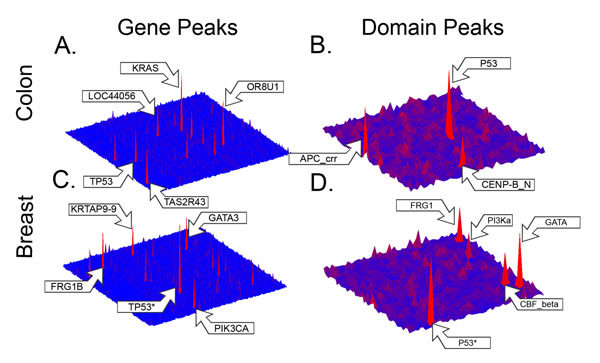
**Gene and domain mutational landscapes for colon and breast cancer** Topographical maps depicting the frequency of somatic mutations in individual genes (1A and 1C) and domains (1B and 1D) from studies of 100 colon adenocarcioma and 522 breast invasive carcinoma patients. Each gene or domain is represented by a single point, and the heights of the peaks on the maps are proportional to the length normalized frequencies of somatic mutations occurring in each gene or domain. The “*” next to the arrow for the P53 domain peak in breast cancer (1D) denotes that the height of this peak was reduced to better show the landscape for the other domains.

Similar to the Wood *et al.* study [2], we plotted the frequencies of colon cancer mutations for individual genes onto a two-dimensional map where each gene is represented as a square of arbitrary size in a grid with coordinates in the x-y axes (Figure 1A). The heights of the peaks (z-axis) on the map are proportional to the frequency of somatic mutations occurring in each gene normalized by the length of the representative protein (i.e. the longest protein isoform) encoded by the gene. This map reveals the overall mutational landscape of genes mutated in colon cancer patients. In comparison to the Wood *et al.*study, we also found a handful of highly mutated gene peaks, including peaks in *KRAS* and *TP53* (Figure 1A) and significant mutation frequency in *APC* (Table 2). Also similar to the Wood *et al.* mutational landscape, we found that the overall gene-based mutational landscape was dominated by a much larger number of lower mutation frequency gene hills. Figure 1C shows the gene-based landscape for breast cancer, revealing a similar topography of mountains and hills. Peaks for the *TP53* gene and *PIK3CA* gene were shared with the Wood *et al*. landscape for breast cancer.

**Table 2:** Selected genes highly mutated in colon cancer tumor genomes

Gene	Protein Accession	Mutations	Protein Length
APC	NP_001120982	76	2843

BRAF	NP_004324	14	766

FBXW7	NP_361014	21	745

KRAS	NP_203524	30	189

LOC440563	NP_001130033	33	293

NRAS	NP_002515	7	189

OR8U1	NP_001005204	48	309

TAS2R19	NP_795369	22	299

TAS2R30	NP_001091112	28	319

TAS2R31	NP_795366	31	309

TAS2R43	NP_795365	36	309

TP53	NP_000537	31	393

Selected list of significantly mutated genes as identified by using local false discovery rate (LFDR) (threshold < 0.1) of the length normalized mutation frequencies for all genes. Mutation counts are the total counts of somatic mutations identified in 100 colon cancer patients falling inside the protein coding region of the gene. Protein length is the number of amino acids in the representative protein isoform for the corresponding gene.

We adapted the local false discovery rate analysis from Efron *et al. *[21] to identify genes and domains with significant mutation frequency. We expected these regions to contain driver mutations under the assumption that non-functional, or passenger, mutations would be uniformly distributed throughout the genome. We also normalized the mutation counts by the representative protein length for genes and by the cumulative domain length for domains, to control for the assumption that longer regions should contain more mutations. This also ensures that the domain landscape is not biased towards more frequently occurring domains. Using a local false discovery rate threshold of 0.1, we identified 154 genes with significant, length normalized mutation frequencies in colon cancer tumor samples (see Additional file 2), and 151 such genes in the breast cancer set (see Additional file 3). The top normalized mutation frequencies occurred in *KRAS*, *OR8U1* and *TAS2R43*(Table 2), with significant mutation frequency for other genes with well-known relevance to colon cancer including *TP53*, *APC* and *BRAF *[15]. In addition, we identified significant mutation frequencies in six CAN genes previously identified in the Sjöblom and Wood studies: *APC*, *KRAS*, *TP53*, *FBXW7*, *SMAD4* and *GRID1.* Of the top five ranked CAN genes, four ranked in the top 20 highest mutation frequency genes in our study, and only *PIK3CA* did not achieve significance despite having nine mutations. However, the PI3K_p85B domain located within the *PIK3CA* gene was found to have significant mutation frequency. Two other CAN genes, *SMAD2* and *SMAD3*, also did not have significant mutation frequency at gene level, but did have significant mutation frequency within the MH2 domain contained within each gene.

### Domain mutation landscapes

As we did for the mutation frequencies for individual genes, we constructed the domain mutational landscape maps by plotting the domain mutation frequencies onto two-dimensional maps where each domain, not gene, is represented as a square of arbitrary size in a grid with coordinates in the x-y axes. The domain peak heights correspond to the mutation frequencies for individual domains containing mutations in the colon (Figure 1B) and breast cancer (Figure 1D) sets, respectively. The count of mutations in each domain was normalized by the cumulative length of all occurrences of the domain from all previously identified representative proteins in the genome. Again using a local false discovery rate threshold of 0.1, we identified 45 domains with significant, length normalized mutation frequencies (see Additional file 4) in the colon cancer set, and 41 such domains in the breast cancer set (see Additional file 5). We found the domain mutation landscape for colon cancer to be dominated by a few peaks corresponding to the P53, APC_crr and CENP-B_N domains, but also to contain a much larger number of smaller domain hills.

**Figure 2:**
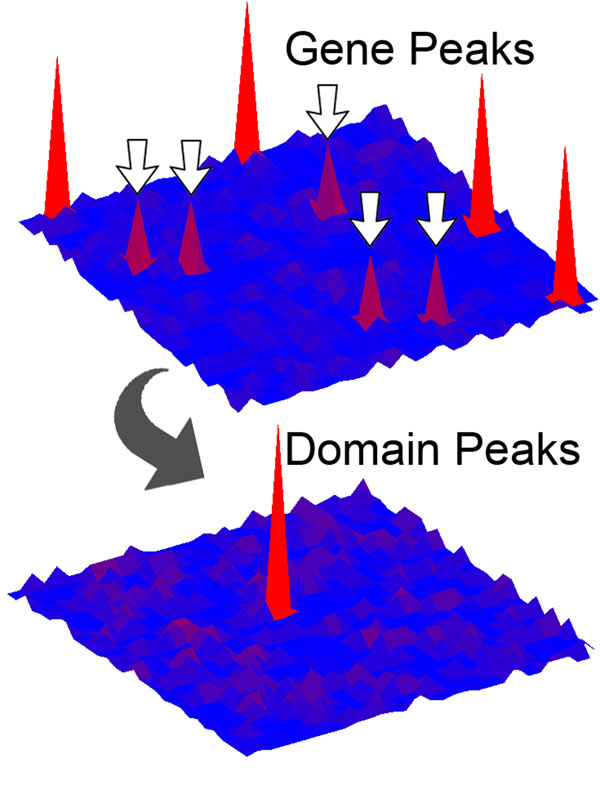
**Domain peaks derived from genes with low mutation frequencies** Depiction of the gene and domain landscape topographies corresponding to an instance where the individual genes contributing mutations to a shared domain do not achieve significance, yet the shared domain aggregates enough mutations to achieve significance. Top map – arrows point to genes with non-significant mutation frequencies. Bottom map – domain peak aggregates enough mutations to be significant.

**Figure 3:**
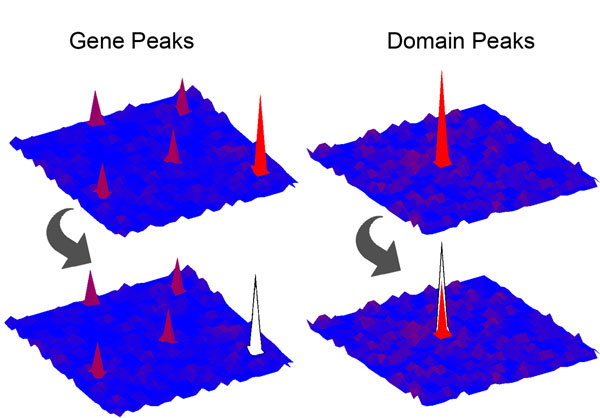
**Domain peaks retaining mutations after the removal of mutations from gene peaks** Depiction of the gene and domain landscape topographies corresponding to an instance where multiple genes contribute mutations to a shared domain, yet the removal of mutations from a significantly mutated gene peak leaves a significant number of mutations in the shared domain. Left side – top map shows the significant gene peak in the lower right corner of the map, bottom map shows the gene peak removed. Right side – top map shows the original domain peak, bottom map shows the domain peak with a significant number of mutations even after the removal of mutations from a significant gene peak.

Construction of both the gene and domain mutational landscapes enabled us to identify a large number of significantly mutated domain peaks that are formed in a variety of ways. Some of our top domain peaks receive most or all of their mutations from gene peaks in the gene landscape (Table 3). For example, in the colon cancer set, P53 and APC_crr receive nearly all of their mutations from the *TP53* and *APC* genes, both of which appeared as gene peaks in our gene landscape. However, some domain peaks reached significance by aggregating mutations from genes that did not individually contain significant numbers of mutations, a graphical depiction of which can be found in Figure 2. One domain peak exhibiting this characteristic was CENP-B_N, which aggregated mutations from genes that were not considered to be significant peaks in the gene landscape, *TIGD7 *and* JRKL*. Comparison of the gene and domain landscapes also enabled us to identify a number of instances where a given domain peak retained mutations even after the removal of mutations occurring in significant gene peaks (see Figure 3).

**Table 3:** Selected domains highly mutated in colon cancer tumor genomes

Name	Accession	Mutations	Cumulative Domain Length	Genes (Number of Mutations)
APC_basic	PF05956	5	356	APC (5)

APC_crr	PF05923	8	176	APC (8)

CortBP2*	PF09727	9	726	CTTNBP2NL (1), CTTNBP2 (2), FILIP1 (5), FILIP1L (1)

MH2	PF03166	14	731	SMAD4 (9), SMAD9 (2), SMAD2 (1), SMAD3 (1), GARS (1)

Miro	PF08477	77	5861	KRAS (28), NRAS (7), RAB27B (2), RAB11B (2), RABL3 (2) + 46 genes with 36 additional mutations

MutS_IV	PF05190	6	380	MSH4 (2), MSH6 (2), MSH5(1), MSH2 (1)

PI3K_p85B	PF02192	4	231	PIK3CA (4)

P53	PF00870	28	390	TP53 (27), TP63 (1)

TAS2R	PF05296	132	6557	TAS2R43 (36), TAS2R31 (31), TAS2R30 (25), TAS2R19 (21) + 19 genes with 27 additional mutations

WAP	PF00095	6	299	WFDC8 (3), SLPI (1), WFDC5 (1), KAL1 (1)

Selected list of significantly mutated domains as identified by LFDR (threshold < 0.1) of the mutation frequencies normalized by the cumulative domain length for all domains. The domain name and Pfam domain accession are provided. Mutation counts are the total counts of somatic mutations identified in 100 colon cancer patients which occur inside the associated protein domain region. Cumulative domain length is the cumulative amino acid length of all occurrences of a domain within all representative proteins for genes containing somatic mutations. The genes and associated mutation counts are listed for each gene containing mutations in the domain. The “*” next to the CortBP2 domain denotes that the domain did not reach the threshold for significant mutation frequency, but ranked in the top 75 highest mutation frequency domains.

### Comparison of colon and breast cancer landscapes

**Figure 4:**
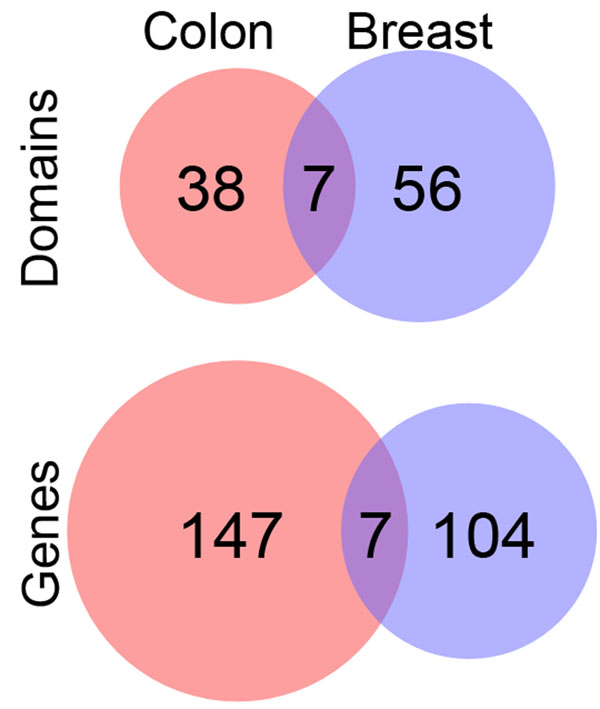
**Shared gene and domain peaks in colon and breast cancer landscapes** Venn diagram illustrating the proportion of overlap between significantly mutated genes and domains in colon and breast cancer.

Using our approach, we found several gene (Additional file 1 – Table S2) and domain peaks (see Additional file 1 – Table S3) in common between the colon and breast cancer gene landscapes. The genes *TP53*, *KRAS*, *CELA1*, *SERTAD3*, *HIST1H1C*, *DCAF4L2* and *BCL2L11 *formed peaks in the mutational landscapes for both the colon and breast cancer sets. In addition, the domains P53, PI3K_p85B, bZIP_1, bZIP_2, IL8, LSM and S_100 formed domain peaks in both cancer types. A Venn diagram (Figure 4) shows the counts of significant gene and domain peaks for both cancer types. We found that the percentage of peaks at the domain level shared between the two cancer types was higher than the percentage of peaks shared at the gene level. For example, while 4.5% (7 out of 154) of the colon cancer gene peaks are shared with breast cancer, approximately 15% (7 out of 45) of the colon cancer domain peaks are shared with breast cancer. We also checked if any of the shared domain peaks were contained within shared gene peaks. Only one of the domain peaks, P53, occurred within a shared gene peak, *TP53*.

### GO term enrichment analysis

To determine functions overrepresented in our sets of significant gene and domain peaks in comparison to genes and domains not reaching significance, we first obtained all available Gene Ontology term (GO term) annotations for all genes and domains containing somatic mutations in our colon and breast cancer sets. A subsequent enrichment analysis of GO terms annotated to gene peaks in our colon cancer landscape revealed an overrepresentation of genes annotated with GO terms related to signal transduction, kinase activity, DNA damage response and the regulation of apoptosis. A list of the top overrepresented GO terms from the enrichment analysis of gene peaks for colon and breast cancer can be found in Additional file 1 – Table S4 and Table S5 respectively. A similar analysis of GO terms annotated to colon cancer domain peaks revealed an overrepresentation of domains annotated with GO terms related to DNA binding, DNA repair, enzyme regulator activity and other cancer-related functional terms such as signal transduction and kinase regulator activity. The complete lists of overrepresented GO terms from the enrichment analysis of domain peaks for colon and breast cancer can be found in Additional file 1 Table_S4 and Table_S5, respectively.

## Discussion

Previous studies by Sjöblom *et al.* and Wood *et al.* identified significant clustering of mutations in the “genomic landscapes” of human breast and colorectal cancers. Despite the need of larger samples to reach more accurate conclusions [1,19,20], these early studies demonstrated the potential of genome-wide studies to capture decades of research into the association of individual genes to cancer in one study. However, due to the rarity of mutations in the gene hills, the authors concluded that these less frequently mutated genes might be better studied within their pathway contexts to elucidate their functional roles in cancer. Today, it is still a major challenge for genome-wide studies of somatic mutations in cancers to identify rare somatic mutations, those gene mutations occurring in a low percentage of tumor samples, that still contribute to cancer initiation and progression.

By mapping mutations not only to the genes, but also to the individual domains they occurred in, we were able to construct the mutational landscapes for both genes and domains for 100 colon cancer patients (Figure 1A and 1B). We also constructed the gene and domain mutational landscapes for 522 breast cancer patients (Figure 1C and 1D) for comparison to another cancer type. Mapping the mutations to specific domains had the advantage of adding the critical functional context necessary for explaining how the mutations potentially contribute to disease. While a relatively small number of significantly mutated domains were shared in both the colon and breast cancer patients, the method also shows the potential of the domain landscape to find commonalities between different cancers at the functional level that might not be apparent at the gene level. Construction of the domain landscape also revealed many properties that are not apparent from traditional gene-based analyses by examining the individual contributions of mutations from distinct genes that fall within a shared domain. These properties include expected instances where a highly mutated gene contained a highly mutated domain, but also unexpected instances where a shared domain is highly mutated, but the individual genes are not, or even where after the removal of mutations from highly mutated genes, some genes still contain mutations within the shared domain. Examination of the domain landscape also revealed instances where all the genes contributed mutations relatively equally to the domain, and where only one or two genes contributed the majority of mutations. We also found instances where highly mutated domains are shared by genes in the same family, and by genes from different families.

Comparison of our gene-based landscape for colon cancer to the landscape constructed by Wood *et al.* revealed similar topographies: a few highly mutated gene mountains along with a much larger number of still significantly mutated gene hills. There was a relatively small overlap between the 154 genes identified by our study and the 140 CAN genes; only six genes were found to be significant in both studies. As noted, the two studies also used different tumor samples and different statistical models to determine significant mutation frequencies. Yet, despite these differences, four of the top five colorectal CAN genes (*APC*, *KRAS*, *TP53* and *FBXW7*) ranked in the top twenty genes with the highest normalized mutation frequency, and the fifth top CAN gene (*PIK3CA*) was identified to have a significantly mutated domain. We also identified seven genes with significant mutation frequency from the Cancer Gene Census [15] list known to have somatic mutations in colorectal cancers including the top five CAN genes, *NRAS* and *BRAF*. A GO term enrichment analysis of all 154 significantly mutated genes in our study identified enrichment in many biological processes and molecular functions known to be disrupted in cancer development including signal transduction, regulation of apoptosis, regulation of cell proliferation and DNA damage response.

Our analysis of the gene landscape resulted in the re-identification of genes with known cancer association and confirmation on enrichment of genes involved in processes critical to cancer development, which validates that our method can identify significantly mutated genes relevant to cancer, and also provides evidence that the method can be applied to other specified regions within the genome, including domain regions. The main focus of this study, however, was the construction of the domain mutational landscape for colon cancer and its comparison with the gene-based mutational landscape. In total, we identified 45 domains with significant mutation frequency in the colon tumor samples. Again, the landscape was characterized by mountains and hills, similar to that of the gene-based landscape, with the highest peaks in the P53, APC_crr and CENP-B_N domains. The CENP-B_N domain, a known DNA-binding domain [22], receives mutations from the *TIGD7* and *JRKL* genes. Although *TIGD7* and *JRKL* are both homologs of the Jrk “jerky” gene associated with epilepsy in mice [23], they do not have known relevance in cancer development. The peaks for P53 and APC_crr were not surprising due to the well-known tumor suppressing functions of the genes containing the domains, *TP53* and *APC*, respectively. However, mapping mutations to the individual domains illustrates the value of our domain-centric method to provide the essential functional context to explain the role the mutations in cancer development. The GO term enrichment analysis for significantly mutated domains confirmed enrichment of significantly mutated domains with functions important to cancer development including kinase activity, DNA binding and repair, and signal transduction.

Our study of the domain landscape of cancer mutations also highlights the relevance of considering the modularity of the proteins when studying somatic mutations. Is the whole protein responsible for the disruption that promotes tumor growth, or are only some of the functional units of the proteins relevant? For instance, the P53 domain, also known as the P53 DNA-binding domain, contains over 90% of the known *TP53* mutations [24], even though the P53 DNA-binding domain covers approximately half of the P53 protein (193 of 393 amino acids). In our study, 27 of the 31 mutations in the P53 protein occurred within the P53 DNA-binding domain. Mutation within the domain has been shown to have multiple detrimental effects including reduced DNA binding affinity, protein misfolding, protein instability and loss of ability to oligomerize (reviewed in [25]). The *APC* gene contains seven repeats of the APC_crr domain that bind to the Arm domains of the beta-catenin protein in addition to thirteen other distinct domains [26]. Truncating mutations mainly within the region of the protein containing the second and third repeats of the APC_crr domain, also referred to as the “mutation cluster region”, are known to eliminate *APC*’s ability to bind and down-regulate beta-catenin, critically impairing its function as a tumor suppressor gene in the Wnt signalling pathway [26,27].

**Figure 5:**
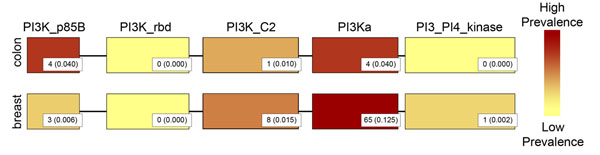
**Comparison of mutation prevalence in PIK3CA domains from colon and breast cancer** Depiction of the mutation prevalence in colon and breast cancer for domains occurring on the PIK3CA gene. Each box represents a distinct domain from the PIK3CA gene. The color of the domain reflects the mutation prevalence for the domain – a mutation prevalence color scale is shown on the right. The mutation prevalence is calculated as the number of mutations occurring in the domain divided by the number of patients in either the colon (100) or breast (522) cancer sets. Each domain is labelled with the count of mutations found within the domain in the PIK3CA gene, with the mutation prevalence in parenthesis.

Despite not reaching significance at the gene level in our colon cancer mutation set, the *PIK3CA* gene ranked in the top five highest, normalized mutation frequencies in the breast cancer set (see Additional file 3), and was also a top colorectal CAN gene in the Wood et al. study. *PI3KCA* functions in signal transduction pathways to mediate signalling for processes such as cell growth and survival, and has been found to be oncogenic in several different cancer types [28]. *PIK3CA* contains a total of five domains, so we compared the domain peaks identified by our method to the domains identified with high mutation prevalence, a measure commonly applied to identify genes mutated in a high percentage of patients. We found that while the PI3K_p85B domain, which is responsible for binding the PI3K p85 subunit to form a heterodimer [29], was identified as a significant domain peak in both cancer types, the domain only had a high mutation prevalence (threshold of 0.04) in the colon cancer set (Figure 5). We also did not find significant mutation frequency or high mutation prevalence in the PI3K_rdb, RAS-binding domain, or in the PI3K_C2 domain, which contains signals for the cellular localization of the PIK3CA protein [30]. The final two domains in the gene, the PI3Ka helical domain and the PI3_PI4_kinase domain, contain known somatic missense mutation hotspots in a variety of cancer types including colon and breast cancer [31]. Only the PI3Ka helical domain had significant mutation frequency and high prevalence in the breast cancer dataset. The PI3Ka domain did not reach significant mutation frequency in the colon cancer set. We also found few mutations from either cancer set in the PI3_PI4_kinase domain, however, the C-terminal region of the domain is believed to be partially disordered [32], likely preventing alignment of the domain model to that region. Therefore, the domain did not pick up mutations in the hotspot.

Together, these examples demonstrate both the advantages and a potential drawback for our domain-based approach. While the traditional, gene centric view of mutation does not consider the location of mutations within the *PIK3CA* gene, our domain-centric approach captures the functional modularity of protein domains and enables us to reveal specific domains critical to the cancer development process. Our approach also identifies domains with significant mutation frequency that might be missed by approaches based on mutation prevalence, as illustrated by the identification of significant mutation frequency in the PI3K_p85B domain in breast cancer patients. Yet, the power of our approach is derived from aggregating mutations from all genes containing a particular domain, therefore currently restricting our method to identifying significant mutation frequency inside domain regions. More work will be needed to extend the scope of our approach to other regions of the genome.

Comparison of the gene and domain landscapes also enabled us to identify a small number of domains, seven in total, which retained mutations even after the removal of mutations contributed from significantly mutated genes. The WAP domain in particular retained a significant number of mutations aggregated from the *WFDC5*, *SLPI* and *KAL1* genes even after the removal of mutations from the significantly mutated *WFDC8* gene. The WAP, whey acidic protein-type, domain contains four disulfide bonds at its core, characteristic of genes with protease inhibitor activity [33]. *WFDC8* has no known association to cancer, however, *WFDC5* has been shown to be upregulated in genes undergoing P53 induced apoptosis [34], and *SLPI* has been shown to promote malignancy in a lung cancer cell line due to its protease inhibitor function [35]. In addition, mutations in *KAL1* are responsible for Kallmann syndrome [36]. Therefore, because of the known cancer and disease relevance of mutations in the WAP domain of other genes, the presence of mutations in the WAP domain of *WFDC8* encourage further study of the role of *WFDC8* in colon cancer development.

The examples discussed above, in which significant domain peaks correspond to at least one significant gene peak only constitute 14 of the 45 significantly mutated domains from the colon tumor set. The other 31 domain peaks correspond to genes without significant mutation frequencies which are undetected in the gene landscape. Because these domains do not occur in significantly mutated genes, they would likely not be found by traditional, gene-centric studies, but may reveal the disruption of potentially critical functional mechanisms within the cancer tissues. One of these peaks corresponds to the cortactin-binding protein-2 domain, CortBP2, that was mutated in four genes, *CTTNBP2NL* (1 mutation), *CTTNBP2* (2 mutations), *FILIP1* (5 mutations), and *FILIP1L* (1 mutation). Interestingly, *FILIP1L* is a highly conserved protein known to inhibit proliferation and migration and increase apoptosis in endothelial cells [37]. This anti-angiogenic protein acts as a tumor suppressor and its loss of function has been implicated in ovarian cancer, head and neck squamous cell carcinoma and oligodendrogliomas [38,39]. While the mutation frequency for the *FILIP1L* gene was not significant in our study, CortBP2 ranked in the top 75 domains with the highest mutation frequency, suggesting a novel role in colon cancer development for *FILIP1L* and the other genes containing mutations in the CortBP2 domain. As with any *in silico* analysis, however, the identification of domains and genes with suspected roles in cancer development can only generate new hypotheses that must ultimately be experimentally validated.

## Conclusions

New methods are critically needed to distinguish mutations that drive tumor initiation and development from the millions of variants being identified in current large-scale tumor sequencing projects. Our novel, domain mutational landscape approach for identifying potential driver mutations in significantly mutated domains reveals many properties that traditional gene landscapes cannot reveal while also adding the functional context necessary for understanding how individual mutations contribute to cancer development. We also compared the mutational landscapes for breast and colon cancer, demonstrating the potential for the domain landscape to identify functional similarities among different cancer types. Determining which mutations are most important for tumorigenesis will shed new light on the selective pressures experienced during the process and will ultimately provide a new set of gene and domain targets for drug development.

## Methods

### Cancer mutation datasets

Controlled access, whole-exome mutation data for 100 colon adenocarcinoma patients and 522 breast invasive carcinoma patients were downloaded from the TCGA Data Portal (http://tcga-data.nci.nih.gov/tcga/) using the mutation files from the ucsc.edu_COAD.IlluminaGA_DNASeq.Level_2.1.0.0 and ucsc.edu_COAD.SOLiD_DNASeq.Level_2.1.0.0 directories downloaded on September 13^th^, 2011 and the genome.wustl.edu_BRCA.IlluminaGA_DNASeq.Level_2.3.0.0 directory downloaded on September 21^st^, 2011 respectively. The individual patient records listed both somatic and germline SNVs and short insertions and deletions (indels) in addition to the tumor and matched normal tissue genotypes for each mutation. The patient records were filtered to remove germline mutations and mutations that did not pass the quality control filter. A union set of somatic SNVs and indels was created for each cancer type from the individual patient records by identifying all somatic mutations present in at least one patient. The genotypes of all patients were then examined for each mutation in the union set to count the number of times the mutation occurred somatically in the patient population. The ANNOVAR program was used to filter out mutations present in dbSNP (release 130) in order to remove mutations known to be polymorphic, therefore unlikely to be cancer driver mutations [40]. ANNOVAR was also used to annotate the mutations within protein coding regions with their associated effects. SNVs were classified as either causing (nonsynonymous SNVs) or not causing (synonymous SNVs) amino acid changes, or either causing the gain (stop-gain) or loss (stop-loss) of a stop codon. Insertions and deletions were classified to either cause (frameshift) or not cause (nonframeshift) a shift in the reading frame. Synonymous SNVs were filtered out of the union set of mutations as they were assumed to be unlikely to affect cancer development. Somatic mutation counts after annotation and filtering are provided for colon (Table 1) and breast cancer (see Additional file 1 – Table S1).

### **Protein and protein domain datasets**

A set of 33,963 human proteins from the RefSeq database [41] was downloaded via NCBI’s E-utilities service. Multiple sequence alignments for all Pfam [17] protein domains were downloaded from the Conserved Domains Database (CDD) [9], and hidden Markov models (HMMs) were built for the domains using the hmmerbuild tool (HMMER version 2.3.2) [42] using default parameters with the global option. HMMER's hmmpfam tool was then used to search for complete Pfam domains in all RefSeq proteins using an E-value cutoff of 0.001. Of the 11,912 Pfam domains, 4,265 mapped to at least one Refseq protein.

### CAN gene datasets

The sets of breast and colorectal cancer candidate genes were downloaded from the Supporting Online Materials section of the Wood *et al.* study, tables S4A and S4B.

### Mapping mutations to individual proteins and domains

All somatic, protein coding mutatons were mapped to individual proteins using ANNOVAR’s exonic variant annotations via the RefSeq transcript accession numbers corresponding to our RefSeq protein accessions [40]. Mutations were mapped to specific protein domains using the hmmpfam alignment output. The method for mapping mutations to their domain positions was previously described for our Domain Mapping of Disease Mutations (DMDM) database (http://bioinf.umbc.edu/DMDM) [13]. We defined the “cancer gene set” as the set of all genes containing at least one somatic mutation after filtering of synonymous SNVs and known polymorphisms. For each gene in the cancer gene set, the longest RefSeq protein isoform was then identified, defining the “cancer protein set”. Only mutations in the longest isoform were considered to avoid replicating individual mutations in the DNA across multiple proteins. Insertions and deletions were mapped to proteins and domains using the starting position of the mutation.

### Calculation of normalized mutation frequency

To determine genes and domains frequently mutated in the patient populations, we first obtained the count of somatic mutations from all patients falling within the protein coding region of each gene, defined in the protein cancer set, and within each protein domain. Because longer regions of DNA are generally expected to accumulate more mutations than shorter regions, we then normalized the gene mutation counts by dividing each count by the length of the gene’s corresponding protein in the protein cancer set. Domain mutation counts were normalized by dividing by the cumulative length of all occurrences of the domain within the protein cancer set.

### Calculation of significantly mutated genes and domains

To detect driver mutations in proteins and domains from a background of passenger mutations, we adapted a method used to estimate the local false discovery rate in microarray experiments by Efron *et al. *[21]. We adjusted the mutation frequency by the length of the domain and use the relative frequency as the success probability (p). We normalized p using the signal to noise ratio of the Bernoulli distribution, which results in a normalized score, z, as follows:(1)

We used aheuristic cut-off of 150 amino acids for the minimum protein or domain length to be included in our analysis. We estimated the null distribution using the “locfdr” package from R and applied these statistics to identify all protein and domains with a local false discovery rate < 0.1.

### GO term enrichment analysis

Gene Ontology terms (GO terms) [43] for all human genes were downloaded from the BioMart portal [44], and were mapped to RefSeq proteins by their corresponding gene symbols. GO terms for individual Pfam domains were obtained from the “pfam2go” file on the Gene Ontology website. The annotations contained in the pfam2go file are derived from mappings of Pfam domains to InterPro domains [45], which are manually annotated with GO terms. Many domains still have unknown or unclear function, therefore we were only able to obtain GO term annotations for approximately 40% of the domains containing at least one somatic mutation. To account for differences in the specificity of GO term annotations, each annotated protein and domain was subsequently assigned all GO terms from all possible paths from the root of the ontology (not including the root term itself) to the annotated GO terms using the full GO ontology in OBO v1.2 format. Individual GO terms from the biological process and molecular function ontologies were tested for enrichment in proteins and domains with significant mutation frequencies using the “calculateStatistic” function of the Text::NSP::Measures::2D::Fisher::right Perl module. For each GO term, the counts for the R function corresponded to a 2 x 2 contingency table based on the counts of proteins or domains either assigned the GO term, or not assigned the GO term, and either having a significant or insignificant mutation frequency. Only proteins and domains containing at least one somatic mutation were considered.

## List of abbreviations

CAN genes: candidate cancer genes identified by the Sjöblom* et al*. and Wood *et al*. studies [1,2]; GO: Gene ontology; LFDR: Local false discovery rate; SNV: single nucleotide variant; TCGA: The Cancer Genome Atlas.

## Authors' contributions

NLN and TAP participated in the preparation and analysis of the data and drafting the manuscript. DP developed the algorithm for computing the mutation frequency significance thresholds. MGK conceived of the study, participated in the analysis, and helped to draft the manuscript. All authors read and approved the final manuscript.

## Competing interests

The authors declare that they have no competing interests.

## Supplementary Material

Additional file 1 **Contains supplementary tables S1 to S7** Table S1 – Mutation counts for breast cancer Table S2 – Shared significantly mutated genes in colon and breast cancer Table S3 – Shared significantly mutated domains in colon and breast cancer Table S4 – GO terms enriched in domains significantly mutated in colon cancer Table S5 - GO terms enriched in domains significantly mutated in breast cancer Table S6 – GO terms enriched in genes significantly mutated in colon cancer Table S7 - GO terms enriched in genes significantly mutated in breast cancer Click here for file

Additional file 2 **Top genes highly mutated in colon cancer tumor genomes** Complete list of significantly mutated genes from the colon cancer set. Gene name, representative protein length, and number of mutations are listed. Click here for file

Additional file 3 **Top genes highly mutated in breast cancer tumor genomes** Complete list of significantly mutated genes from the breast cancer set. Gene name, representative protein length, and number of mutations are listed. Click here for file

Additional file 4 **Top domains highly mutated in colon cancer tumor genomes** Complete list of significantly mutated domains from the colon cancer set. Gene name, Pfam domain accession, number of mutations, cumulative domain length, and genes containing mutations in the domain are listed. Click here for file

Additional file 5 **Top domains highly mutated in breast cancer tumor genomes** Complete list of significantly mutated domains from the breast cancer set. Gene name, Pfam domain accession, number of mutations, cumulative domain length, and genes containing mutations in the domain are listed. Click here for file
