# Supplementary material for: Domain landscapes of somatic mutations in cancer
Source: BMC Genomics. 2012 Jun 18;13(Suppl 4):S9. doi: 10.1186/1471-2164-13-S4-S9 (PMC3394412; doi:10.1186/1471-2164-13-S4-S9)
Supplement: Additional file 1 — Contains supplementary tables S1 to S7 Table S1 – Mutation counts for breast cancer Table S2 – Shared significantly mutated genes in colon and breast cancer Table S3 – Shared significantly mutated domains in colon and breast cancer Table S4 – GO terms enriched in domains significantly mutated in colon cancer Table S5 - GO terms enriched in domains significantly mutated in breast cancer Table S6 – GO terms enriched in genes significantly mutated in colon cancer Table S7 - GO terms enriched in genes significantly mutated in breast cancer [file 1471-2164-13-S4-S9-S1.docx]

## Additional file 1

## Table S1 - Mutation counts for breast cancer

Summary of somatic mutations from the exomes of 522 breast cancer tumor genomes. Synonymous SNVs and variants present in dbSNP (release 130) were removed due to their low likelihood of being driver mutations.

| Total patients | 522 |
| --- | --- |
| Total mutations | 25,807 |
| Total nonsynonymous SNVs | 21,203 (82.2%) |
| Total frameshift insertions | 534 (2.1%) |
| Total nonframeshift insertions | 364 (1.4%) |
| Total frameshift deletions | 1,307 (5.1%) |
| Total nonframeshift deletions | 718 (2.8%) |
| Total stop-loss SNVs | 36 (0.1%) |
| Total stop-gain SNVs | 1,645 (6.4%) |
| Mutations at domain regions | 11,694 (45.3%) |
| Average mutations per patient | 49.70 (± 75.0) |
| Number of mutations per patient | 2-831 |

## Table S2 – Shared significantly mutated genes in colon and breast cancer

List of significantly mutated genes occurring in both colon and breast cancer tumor samples as identified by an adapted method used to estimate the local false discovery rate in microarray experiments by Efron *et al.* [21] (threshold < 0.1).

| Gene | Protein Accession | Number of Mutations in  Colon Tumors | Number of Mutations in Breast Tumors |
| --- | --- | --- | --- |
| TP53 | NP_000537 | 31 | 160 |
| KRAS | NP_203524 | 30 | 4 |
| CELA1 | NP_001962 | 10 | 18 |
| DCAF4L2 | NP_689631 | 5 | 7 |
| HIST1H1C | NP_005310 | 3 | 4 |
| SERTAD3 | NP_976219 | 3 | 3 |
| BCL2L11 | NP_619527 | 3 | 3 |

**Table S3 – Shared significantly mutated domains in colon and breast cancer**

List of significantly mutated domains occurring in both colon and breast cancer tumor samples as identified by an adapted method used to estimate the local false discovery rate in microarray experiments by Efron *et al.* [21] (threshold < 0.1).

| Domain | Number of Mutations in Colon Tumors | Number of Mutations in Breast Tumors |
| --- | --- | --- |
| P53 | 28 | 142 |
| IL8 | 5 | 11 |
| bZIP_1 | 6 | 8 |
| bZIP_2 | 6 | 8 |
| PI3K_p85B | 4 | 4 |
| S_100 | 4 | 3 |
| LSM | 3 | 4 |

**Table S4 – GO terms enriched in domains significantly mutated in colon cancer**

List of Gene Ontology terms found to be enriched with significantly mutated domains in colon cancer tumors as identified by local FDR analysis. The significance P-value for each GO term was calculated using Fisher’s exact test.

| GO Accession | P-value | Name | Ontology |
| --- | --- | --- | --- |
| GO:0003677 | 6.57E-04 | DNA binding | molecular function |
| GO:0030234 | 3.60E-03 | enzyme regulator activity | molecular function |
| GO:0007166 | 1.44E-02 | cell surface receptor linked signaling pathway | biological process |
| GO:0006974 | 2.08E-02 | response to DNA damage stimulus | biological process |
| GO:0006259 | 2.46E-02 | DNA metabolic process | biological process |
| GO:0008252 | 2.52E-02 | nucleotidase activity | molecular function |
| GO:0035383 | 2.52E-02 | thioester metabolic process | biological process |
| GO:0061134 | 2.52E-02 | peptidase regulator activity | molecular function |
| GO:0019207 | 2.52E-02 | kinase regulator activity | molecular function |
| GO:0050877 | 2.52E-02 | neurological system process | biological process |
| GO:0016289 | 2.52E-02 | CoA hydrolase activity | molecular function |
| GO:0052813 | 2.52E-02 | phosphatidylinositol bisphosphate kinase activity | molecular function |
| GO:0007606 | 2.52E-02 | sensory perception of chemical stimulus | biological process |
| GO:0042379 | 2.52E-02 | chemokine receptor binding | molecular function |
| GO:0005125 | 2.52E-02 | cytokine activity | molecular function |
| GO:0007600 | 2.52E-02 | sensory perception | biological process |
| GO:0016835 | 2.52E-02 | carbon-oxygen lyase activity | molecular function |
| GO:0000975 | 2.52E-02 | regulatory region DNA binding | molecular function |
| GO:0090304 | 3.25E-02 | nucleic acid metabolic process | biological process |
| GO:0051716 | 3.25E-02 | cellular response to stimulus | biological process |
| GO:0006281 | 3.48E-02 | DNA repair | biological process |
| GO:0044260 | 3.70E-02 | cellular macromolecule metabolic process | biological process |
| GO:0007165 | 4.80E-02 | signal transduction | biological process |
| GO:0015631 | 4.97E-02 | tubulin binding | molecular function |
| GO:0001067 | 4.97E-02 | regulatory region nucleic acid binding | molecular function |
| GO:0004857 | 4.97E-02 | enzyme inhibitor activity | molecular function |

## Table S5 – GO terms enriched in domains significantly mutated in breast cancer

List of Gene Ontology terms found to be enriched with significantly mutated domains in breast cancer tumors as identified by local FDR analysis. The significance P-value for each GO term was calculated using Fisher’s exact test.

| GO Accession | P-value | Name | Ontology |
| --- | --- | --- | --- |
| GO:0003677 | 4.11E-09 | DNA binding | molecular function |
| GO:0010468 | 1.28E-07 | regulation of gene expression | biological process |
| GO:2001141 | 1.28E-07 | regulation of RNA biosynthetic process | biological process |
| GO:2000112 | 1.28E-07 | regulation of cellular macromolecule biosynthetic process | biological process |
| GO:0001071 | 6.51E-07 | nucleic acid binding transcription factor activity | molecular function |
| GO:0005488 | 2.10E-04 | binding | molecular function |
| GO:0005515 | 3.55E-03 | protein binding | molecular function |
| GO:0003676 | 3.62E-03 | nucleic acid binding | molecular function |
| GO:0003712 | 9.02E-03 | transcription cofactor activity | molecular function |
| GO:0000989 | 1.19E-02 | transcription factor binding transcription factor activity | molecular function |
| GO:0000988 | 1.19E-02 | protein binding transcription factor activity | molecular function |
| GO:0002376 | 1.19E-02 | immune system process | biological process |
| GO:0006355 | 1.50E-02 | regulation of transcription, DNA-dependent | biological process |
| GO:0052742 | 2.17E-02 | phosphatidylinositol kinase activity | molecular function |
| GO:0051259 | 2.17E-02 | protein oligomerization | biological process |
| GO:0043021 | 2.17E-02 | ribonucleoprotein complex binding | molecular function |
| GO:0052813 | 2.17E-02 | phosphatidylinositol bisphosphate kinase activity | molecular function |
| GO:0042379 | 2.17E-02 | chemokine receptor binding | molecular function |
| GO:0005125 | 2.17E-02 | cytokine activity | molecular function |
| GO:0016407 | 2.17E-02 | acetyltransferase activity | molecular function |
| GO:0000975 | 2.17E-02 | regulatory region DNA binding | molecular function |
| GO:0050896 | 2.24E-02 | response to stimulus | biological process |
| GO:0031328 | 4.30E-02 | positive regulation of cellular biosynthetic process | biological process |
| GO:0048522 | 4.30E-02 | positive regulation of cellular process | biological process |
| GO:0051254 | 4.30E-02 | positive regulation of RNA metabolic process | biological process |
| GO:0010604 | 4.30E-02 | positive regulation of macromolecule metabolic process | biological process |
| GO:0042981 | 4.30E-02 | regulation of apoptosis | biological process |
| GO:0009889 | 4.30E-02 | regulation of biosynthetic process | biological process |
| GO:0010557 | 4.30E-02 | positive regulation of macromolecule biosynthetic process | biological process |
| GO:0031326 | 4.30E-02 | regulation of cellular biosynthetic process | biological process |
| GO:0009893 | 4.30E-02 | positive regulation of metabolic process | biological process |
| GO:0001067 | 4.30E-02 | regulatory region nucleic acid binding | molecular function |
| GO:0031323 | 4.30E-02 | regulation of cellular metabolic process | biological process |
| GO:0043068 | 4.30E-02 | positive regulation of programmed cell death | biological process |
| GO:0010556 | 4.30E-02 | regulation of macromolecule biosynthetic process | biological process |
| GO:0010628 | 4.30E-02 | positive regulation of gene expression | biological process |
| GO:0048518 | 4.30E-02 | positive regulation of biological process | biological process |
| GO:0009891 | 4.30E-02 | positive regulation of biosynthetic process | biological process |
| GO:0001727 | 4.30E-02 | lipid kinase activity | molecular function |
| GO:0031325 | 4.30E-02 | positive regulation of cellular metabolic process | biological process |
| GO:0009207 | 4.30E-02 | purine ribonucleoside triphosphate catabolic process | biological process |

## Table S6 – Top GO terms enriched in genes significantly mutated in colon cancer

List of Gene Ontology terms found to be enriched with significantly mutated genes in colon cancer tumors as identified by local FDR analysis. The significance P-value for each GO term was calculated using Fisher’s exact test.

| GO:0038023 | 1.86E-23 | signaling receptor activity | molecular function |
| --- | --- | --- | --- |
| GO:0004888 | 1.79E-13 | transmembrane signaling receptor activity | molecular function |
| GO:0043065 | 7.55E-10 | positive regulation of apoptosis | biological process |
| GO:0048522 | 5.23E-09 | positive regulation of cellular process | biological process |
| GO:0005515 | 2.85E-08 | protein binding | molecular function |
| GO:0004675 | 1.54E-07 | transmembrane receptor protein serine/threonine kinase activity | molecular function |
| GO:0050789 | 2.42E-07 | regulation of biological process | biological process |
| GO:0004674 | 6.97E-07 | protein serine/threonine kinase activity | molecular function |
| GO:0042981 | 1.01E-06 | regulation of apoptosis | biological process |
| GO:0043067 | 1.15E-06 | regulation of programmed cell death | biological process |
| GO:0012502 | 1.51E-06 | induction of programmed cell death | biological process |
| GO:0007166 | 1.57E-06 | cell surface receptor linked signaling pathway | biological process |
| GO:0051716 | 1.84E-06 | cellular response to stimulus | biological process |
| GO:0005102 | 2.62E-06 | receptor binding | molecular function |
| GO:0019199 | 2.67E-06 | transmembrane receptor protein kinase activity | molecular function |
| GO:0090100 | 3.03E-06 | positive regulation of transmembrane receptor protein serine/threonine kinase signaling pathway | biological process |
| GO:0007165 | 3.24E-06 | signal transduction | biological process |
| GO:0007167 | 5.64E-06 | enzyme linked receptor protein signaling pathway | biological process |
| GO:0030234 | 1.48E-05 | enzyme regulator activity | molecular function |
| GO:0051239 | 1.69E-05 | regulation of multicellular organismal process | biological process |
| GO:0051048 | 1.84E-05 | negative regulation of secretion | biological process |
| GO:0032502 | 1.91E-05 | developmental process | biological process |
| GO:0042127 | 2.30E-05 | regulation of cell proliferation | biological process |
| GO:0007606 | 2.49E-05 | sensory perception of chemical stimulus | biological process |
| GO:0002700 | 3.28E-05 | regulation of production of molecular mediator of immune response | biological process |
| GO:0002706 | 3.55E-05 | regulation of lymphocyte mediated immunity | biological process |
| GO:0048523 | 4.06E-05 | negative regulation of cellular process | biological process |
| GO:0006468 | 5.48E-05 | protein phosphorylation | biological process |
| GO:0048519 | 6.81E-05 | negative regulation of biological process | biological process |
| GO:0050877 | 6.93E-05 | neurological system process | biological process |
| GO:0031401 | 8.18E-05 | positive regulation of protein modification process | biological process |
| GO:0007600 | 9.53E-05 | sensory perception | biological process |
| GO:0019207 | 1.03E-04 | kinase regulator activity | molecular function |
| GO:0002703 | 1.07E-04 | regulation of leukocyte mediated immunity | biological process |
| GO:0042221 | 1.30E-04 | response to chemical stimulus | biological process |
| GO:0001934 | 1.39E-04 | positive regulation of protein phosphorylation | biological process |
| GO:0050864 | 1.62E-04 | regulation of B cell activation | biological process |
| GO:0019222 | 1.62E-04 | regulation of metabolic process | biological process |
| GO:0050906 | 1.67E-04 | detection of stimulus involved in sensory perception | biological process |
| GO:0032147 | 1.74E-04 | activation of protein kinase activity | biological process |
| GO:0007568 | 1.80E-04 | aging | biological process |
| GO:0001817 | 2.22E-04 | regulation of cytokine production | biological process |
| GO:0051241 | 2.34E-04 | negative regulation of multicellular organismal process | biological process |

## Table S7 – Top GO terms enriched in genes significantly mutated in breast cancer

List of Gene Ontology terms found to be enriched with significantly mutated genes in breast cancer tumors as identified by local FDR analysis. The significance P-value for each GO term was calculated using Fisher’s exact test.

| GO:0048522 | 1.83E-18 | positive regulation of cellular process | biological process |
| --- | --- | --- | --- |
| GO:0051716 | 1.92E-23 | cellular response to stimulus | biological process |
| GO:0050789 | 6.64E-21 | regulation of biological process | biological process |
| GO:0008150 | 3.29E-17 | biological process | biological process |
| GO:0005515 | 8.33E-17 | protein binding | molecular function |
| GO:0048518 | 1.22E-15 | positive regulation of biological process | biological process |
| GO:0010468 | 1.51E-15 | regulation of gene expression | biological process |
| GO:2000112 | 1.74E-14 | regulation of cellular macromolecule biosynthetic process | biological process |
| GO:0032502 | 2.73E-13 | developmental process | biological process |
| GO:0019222 | 3.30E-13 | regulation of metabolic process | biological process |
| GO:0009893 | 7.32E-13 | positive regulation of metabolic process | biological process |
| GO:0060255 | 9.52E-13 | regulation of macromolecule metabolic process | biological process |
| GO:0050896 | 1.35E-12 | response to stimulus | biological process |
| GO:0031323 | 1.64E-12 | regulation of cellular metabolic process | biological process |
| GO:2001141 | 4.60E-12 | regulation of RNA biosynthetic process | biological process |
| GO:0048856 | 7.43E-12 | anatomical structure development | biological process |
| GO:0031325 | 1.19E-11 | positive regulation of cellular metabolic process | biological process |
| GO:0042981 | 4.65E-11 | regulation of apoptosis | biological process |
| GO:0043065 | 8.39E-11 | positive regulation of apoptosis | biological process |
| GO:0006355 | 1.04E-10 | regulation of transcription, DNA-dependent | biological process |
| GO:0048523 | 1.15E-10 | negative regulation of cellular process | biological process |
| GO:0070887 | 1.31E-10 | cellular response to chemical stimulus | biological process |
| GO:0031326 | 4.28E-10 | regulation of cellular biosynthetic process | biological process |
| GO:0031328 | 8.06E-10 | positive regulation of cellular biosynthetic process | biological process |
| GO:0009889 | 1.12E-09 | regulation of biosynthetic process | biological process |
| GO:0009891 | 1.52E-09 | positive regulation of biosynthetic process | biological process |
| GO:0043067 | 2.63E-09 | regulation of programmed cell death | biological process |
| GO:0071310 | 4.57E-09 | cellular response to organic substance | biological process |
| GO:0048583 | 4.93E-09 | regulation of response to stimulus | biological process |
| GO:0007165 | 5.29E-09 | signal transduction | biological process |
| GO:0004888 | 5.97E-09 | transmembrane signaling receptor activity | molecular function |
| GO:0009966 | 7.27E-09 | regulation of signal transduction | biological process |
| GO:0030154 | 8.52E-09 | cell differentiation | biological process |
| GO:0010604 | 9.83E-09 | positive regulation of macromolecule metabolic process | biological process |
| GO:0023051 | 1.13E-08 | regulation of signaling | biological process |
| GO:0007169 | 1.96E-08 | transmembrane receptor protein tyrosine kinase signaling pathway | biological process |
| GO:0051254 | 2.84E-08 | positive regulation of RNA metabolic process | biological process |
| GO:0042127 | 3.03E-08 | regulation of cell proliferation | biological process |
| GO:0002221 | 3.24E-08 | pattern recognition receptor signaling pathway | biological process |
